# Microbial experimental evolution as a novel research approach in the Vibrionaceae and squid-*Vibrio* symbiosis

**DOI:** 10.3389/fmicb.2014.00593

**Published:** 2014-12-09

**Authors:** William Soto, Michele K. Nishiguchi

**Affiliations:** ^1^BEACON Center for the Study of Evolution in Action, Michigan State UniversityEast Lansing, MI, USA; ^2^Department of Biology, New Mexico State UniversityLas Cruces, NM, USA

**Keywords:** *Vibrio*, sepiolid squid, cospeciation, experimental evolution, environmental transmission

## Abstract

The Vibrionaceae are a genetically and metabolically diverse family living in aquatic habitats with a great propensity toward developing interactions with eukaryotic microbial and multicellular hosts (as either commensals, pathogens, and mutualists). The Vibrionaceae frequently possess a life history cycle where bacteria are attached to a host in one phase and then another where they are free from their host as either part of the bacterioplankton or adhered to solid substrates such as marine sediment, riverbeds, lakebeds, or floating particulate debris. These two stages in their life history exert quite distinct and separate selection pressures. When bound to solid substrates or to host cells, the Vibrionaceae can also exist as complex biofilms. The association between bioluminescent *Vibrio* spp. and sepiolid squids (Cephalopoda: Sepiolidae) is an experimentally tractable model to study bacteria and animal host interactions, since the symbionts and squid hosts can be maintained in the laboratory independently of one another. The bacteria can be grown in pure culture and the squid hosts raised gnotobiotically with sterile light organs. The partnership between free-living *Vibrio* symbionts and axenic squid hatchlings emerging from eggs must be renewed every generation of the cephalopod host. Thus, symbiotic bacteria and animal host can each be studied alone and together in union. Despite virtues provided by the Vibrionaceae and sepiolid squid-*Vibrio* symbiosis, these assets to evolutionary biology have yet to be fully utilized for microbial experimental evolution. Experimental evolution studies already completed are reviewed, along with exploratory topics for future study.

## THE VIBRIONACEAE

The family Vibrionaceae (Domain Bacteria, Phylum Proteobacteria, Class Gammaproteobacteria) encompass gram-negative chemoorganotrophs that are mostly motile and possess at least one polar flagellum (Farmer III and Janda, 2005; Thompson and Swings, 2006); although, the gut symbiont *Vibrio halioticoli* to the abalone *Haliotis discus hannai* has been described as non-motile (Sawabe et al., 1998). Vibrionaceae are facultative anaerobes, having both respiratory (aerobic and anaerobic) and fermentative metabolisms. Nitrogen fixation and phototrophy have both been reported (Criminger et al., 2007; Wang et al., 2012). Agarases and alginases have been noted from *Vibrio* (Fu and Kim, 2010; Dalia et al., 2014). Most cells are oxidase positive with a dimension 1 μm in width and 2–3 μm in length. Sodium cations are a requirement for growth and survival, but *Vibrio cholerae* and *V. mimicus* are unusually tolerant to low sodium waters. Most species are susceptible to the vibriostatic agent 0/129 (Thompson and Swings, 2006). Vibrionaceae are ubiquitously distributed throughout aquatic habitats, including freshwater, brackish, and marine waters (Madigan and Martinko, 2006). Vibrionaceae have been isolated from rivers, estuaries, lakes, coastal and pelagic oceanic waters, the deep sea, and saltern ponds (Urakawa and Rivera, 2006). Vibrionaceae can also be microbial residents of aquatic animals as either commensals, pathogens, and mutualists (Soto et al., 2010). Bacteria may exist as planktonic free-living cells or as biofilms attached to solid subtrates present in sediments of aquatic habitats or alternatively adhered to floating particulate matter or debris. Vibrionaceae may also form biofilms on the surfaces of animal, algal/phytoplanktonic, protoctistal, or fungal hosts the cells colonize, as this prokaryotic family is quite able to initiate and establish vigorous biofilms on eukaryotic cells and chitin surfaces (e.g., invertebrate exoskeletons and fungal cell walls; Polz et al., 2006, Pruzzo et al., 2008; Soto et al., 2014). Vibrionaceae have also been found to be intracellular inhabitants of eukaryotic microorganisms (Abd et al., 2007). Although as many as eight genera have been assigned to the Vibrionaceae, the two most specious are *Vibrio* and *Photobacterium* (Thompson and Swings, 2006). *Salinivibrio* possesses an unusual ability to grow in a wide range of salinity (0–20% NaCl) and temperature (5–50°C; Ventosa, 2005; Bartlett, 2006). Numerous species in the Vibrionaceae are pathogenic and cause disease in aquatic animals and humans (Farmer III et al., 2005), *V. cholerae* being the most notorious example as the causative agent of cholera (Colwell, 2006). *V. vulnificus* and *V. parahaemolyticus* can also cause severe illnesses in humans as a result of consuming contaminated seafood (Hulsmann et al., 2003; Wong and Wang, 2004). Furthermore, every year *V. harveyi* (Owens and Busico-Salcedo, 2006), *V. anguillarum* (Miyamoto and Eguchi, 1997; Crosa et al., 2006), and *V. parahaemolyticus* (Austin, 2006) cause substantial economic losses to the aquaculture industry worldwide. The genera *Vibrio* and *Photobacterium* include opportunistic pathogens capable of infecting marine animals and humans, and are able to enter preexisting wounds or body openings of especially susceptible hosts that are already ill, stressed, fatigued, or immunocompromised (Urbanczyk et al., 2011). Given the heightened ability of Vibrionaceae to cement themselves to eukaryotic cells through peptide and polysaccharide modification of their exopolysaccharide, lipopolysaccharide, and capsules (Sozhamannan and Yildiz, 2011), the lack of additional human pathogens is curious. Perhaps the reason is foreign extracellular protein and polysaccharide are the same materials the mammalian immune system specifically targets, neutralizes, and removes as non-self antigens with exquisite capacity (Owen et al., 2013). Vibrionaceae have also been recently investigated for the development of probiotics, antimicrobials, and pharmaceutical drugs with potential clinical and economic value for veterinary medicine, animal husbandry, aquaculture, and human health—molecules antagonistic toward cancer cells, fungi, algae, protoctists (a term frequently preferred over protist or protozoan), bacteria, and viruses. Metabolites produced by the Vibrionaceae have also been found to have quorum sensing-disrupting properties against other bacteria, which may open an entire horizon for the advancement of “quorum sensing” antibiotics (i.e., quorum quenching) (Gatesoupe, 1999; Mansson et al., 2011).

## MICROBIAL EXPERIMENTAL EVOLUTION

Conventional evolutionary studies seeking to understand adaptation and speciation implement the comparative or historical approach (e.g., phylogenetics). This approach compares organisms from different environments and attempts to understand the evolutionary processes that may have produced the current distributions and adaptations of descendent populations from ancestral ones (Bennett, 2002). Since this methodology generates informed explanations based on extant organisms retrospectively and with hindsight, it naturally must make numerous assumptions on the evolutionary relationships of the organisms under study and their likely mode of evolution, even when the use of fossil data is available. Experimental evolution, however, allows one to begin with an ancestral population and empirically observe the adaptation and radiation that result in the descendent lineages under different selective regimens. Experimental evolution studies can be implemented under controlled and reproducible conditions to study evolution, usually in the laboratory and on model organisms. Less assumptions in environmental conditions, the selection pressures involved, or in the ancestral and evolving populations are necessary, since there is more control by the investigator (Bennett, 2002). Experimental evolution permits tractability for the study of evolutionary biology by allowing experiments to be manipulated and repeated with replication (Lenski, 1995; Bennett, 2002). Bacteria, including *Vibrio*, are ideal organisms for such studies. For instance, these organisms have short generation times which allow evolution and adaptation to be observable on a human time scale (Lenski, 1995). Microorganisms also usually possess the advantage of achieving large population sizes (>1 × 10^9^ cells/mL in liquid culture) in the environments for which experimental evolution studies are executed, providing ample opportunity for rare beneficial mutations to arise and reach fixation by natural selection. Deleterious mutations are likely to become extinct via purifying selection, since evolution by genetic drift is negligible in huge gene pools. Moreover, a “frozen fossil record” can be generated with bacteria by storing evolving lineages at different evolutionary time points in a -80°C freezer. Hence, one can later compare relative fitness of the ancestral clone with a derived one in novel or ancestral environments (Bennett, 2002; Lenski, 2002). As a result, evolutionary tradeoffs can be measured during the course of adaptation in the novel environment. The -80°C fossil record also permits the determination of the evolutionary episode that a novel adaptive trait first evolved. Likewise, evolution may be “replayed” from various time points to see if subsequent outcomes are contingent on prior genetic changes or previously modified traits (Kawecki et al., 2012; Barrick and Lenski, 2013). Finally, the ancestral and derived bacteria can subsequently be analyzed to observe the exact genetic changes that have occurred and which specific ones are responsible for novel adaptive traits (Lenski, 1995; Bennett, 2002). Experimental evolution is the only direct method for studying adaptation and the genetic changes responsible, which complements genetic, physiological, biochemical, and phylogenetic approaches.

## ATTENUATION AND VACCINE DEVELOPMENT WITH VIBRIOS: INSIGHTS FOR MICROBIAL EXPERIMENTAL EVOLUTION

Microbial experimental evolution is a thrilling sub-discipline of evolutionary biology which has risen in the last twenty to thirty years to address diverse issues (Soto et al., 2010; Conrad et al., 2011). Although initial work largely began with *Escherichia coli* (Lenski et al., 1991), the inclusion of other microbial species has continued to grow. However, despite a few exceptions (Schuster et al., 2010; Soto et al., 2012, 2014), surprisingly little work has been completed to date with members of the Vibrionaceae. Considering the Vibrionaceae possess colossal metabolic, biochemical, ecological, and genetic diversity, the general absence of this bacterial family as an established model in microbial experimental evolution has been heedless. Nonetheless, classical efforts to attenuate pathogenic bacteria for human vaccine development were endeavors analogous to experimental evolution (Kawecki et al., 2012). Virulent bacterial isolates would be repeatedly subcultured under laboratory conditions on growth medium, in tissue/cell culture, or in animal models to introduce random deleterious mutations in the microorganism under study. Alternatively, the microbe would be continuously subjected to chemical or physical mutagens (e.g., ultraviolet light). The exact mutations that occurred and the loci undergoing genetic changes were frequently unknown initially, and attempts to their identification only coming later with additional research (Frey, 2007). For *V. cholerae*, nitrosoguanidine frequently served as a chemical mutagen to induce several attenuating mutations, including auxotrophy (Bhaskaran and Sinha, 1967; Baselski et al., 1978). Although attenuation by random mutagenesis yielded some products that demonstrated promising results in animal models and humans, this approach is less common today (Frey, 2007). The construction of attenuated vibrios containing large targeted deletions of loci known to contribute to virulence is currently more desirable, since microbial reversion to pathogenicity is deemed less probable through this practice. Side effects are also a concern (Honda and Finkelstein, 1979; Cameron and Mekalanos, 2011). *V. cholerae* attenuation by the continual introduction of random mutations, resulting in numerous deleterious genetic lesions across many loci, frequently fails to sufficiently incapacitate virulence (Cameron and Mekalanos, 2011), as the microbe finds alternative ways to thrive and persist in the human host. An evolutionary conclusion coming from vaccine work with *V. cholerae* is that numerous ways of making a successful living exist in a potential host for the genus *Vibrio*; many potential niches exist, as evidenced by the continued ability of *V. cholerae* to initiate successful and alternative symptomatic infections (e.g., reactogenicity) despite the introduction of several deleterious mutations into its genome. An implication of this observation is that vibrios are evolutionarily versatile for host colonization and proliferation. For instance, medical reports exist of *V. cholerae*’s ability to initiate bacteremia, malaise, fever, chills, and skin lesions in humans, even in the absence of a gastrointestinal infection (Ninin et al., 2000). Such symptoms are more typically characteristic of *V. vulnificus* infections and raise the interesting question of whether there may be common virulence factors in *V. cholerae* and other pathogenic vibrios which are overshadowed by the exuberant effect of choleragen. More broadly, determinants and mechanisms responsible for the colonization of host animals (or attachment to eukaryotic cells) by vibrios may possess overlap across diverse interactions (e.g., commensalism, pathogenesis, and mutualism; Hentschel et al., 2000). Hence, microbial selection experiments with vibrios have potential to provide novel insights into evolution of the varied interactions the genus *Vibrio* possesses with its hosts, and vibrio vaccine research is a great repository of information and useful starting point to ask scientific questions, construct hypotheses, and to find focus topics for real world applications and practical value.

## SEPIOLID SQUID-*VIBRIO* SYMBIOSIS: A CASE STUDY FOR MICROBIAL EXPERIMENTAL EVOLUTION WITH THE VIBRIONACEAE

**FIGURE 1:**
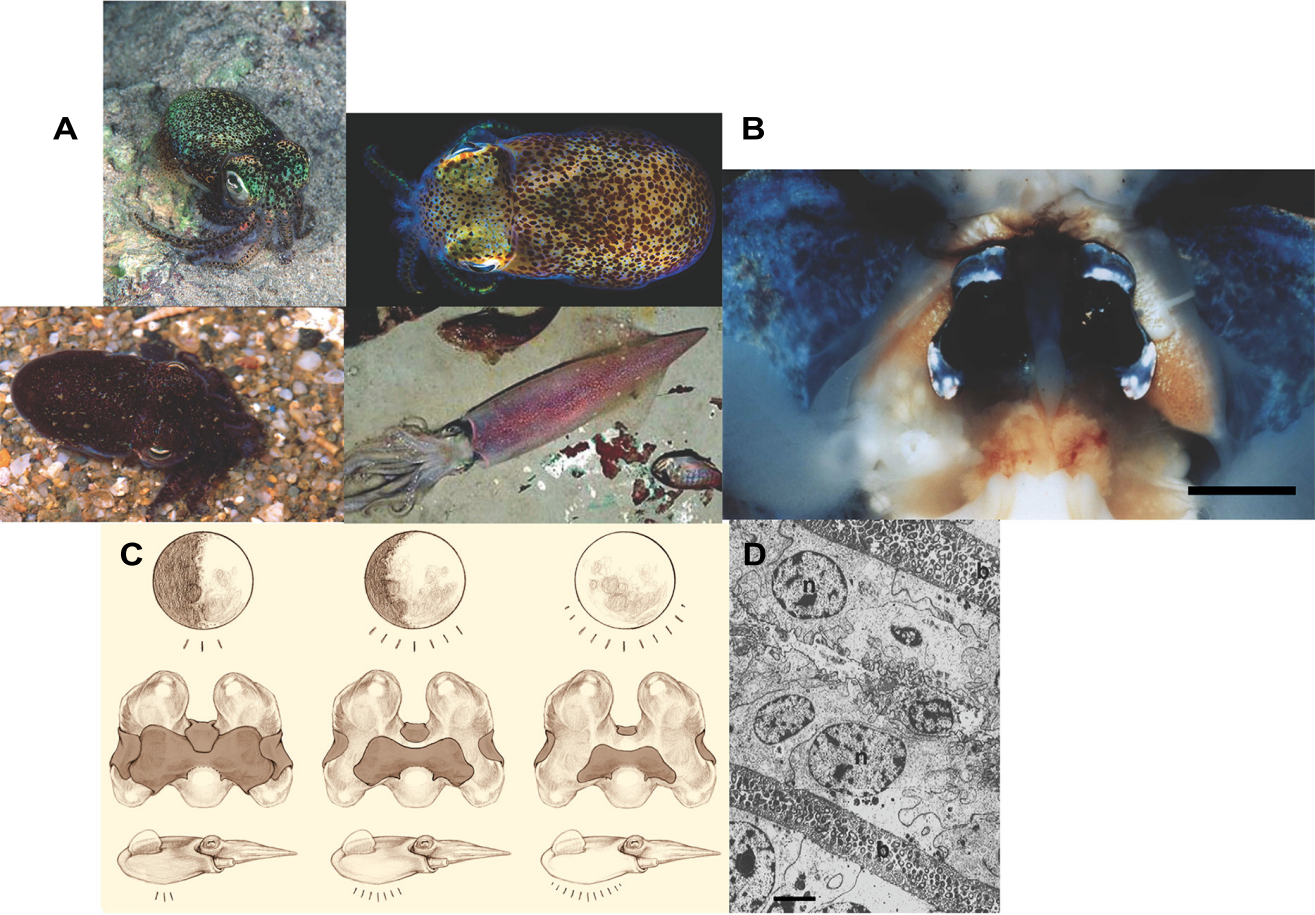
**The sepiolid squid-*Vibrio* symbiosis. (A)** Representative species from the families Sepiolidae and Loliginidae (clockwise from upper left): *Euprymna tasmanica* (Sepiolidae), *E. scolopes* (Sepiolidae), *Photololigo noctiluca* (Loliginidae), and *Sepiola affinis* (Sepiolidae). **(B)** Ventral dissection of *E. scolopes*, showing the bilobed light organ surrounded by the ink sac. Bar = 0.5 cm. **(C)** Diagram how the light organ operates under different phases of the moon. The progressive decrease in shading from left to right symbolizes increased illumination by the light organ. **(D)** A transmission electron micrograph of an area of the epithelium-lined crypts containing symbiotic bacteria: (n) = nucleus of squid cell, (b) = bacteria in crypts (bar = 10 μm). Photo credits: Mark Norman, Mattias Oremstedt (Kahikai), M. K. Nishiguchi, R. Young, S. Nyholm, R. Long, M. Montgomery. Light organ illustration by Robert Long-Nearsight graphics.

As mentioned previously, many members of the Vibrionaceae are able to form associations with eukaryotic hosts, including phytoplankton, protoctists, algae, aquatic fungi, invertebrates, fishes, and aquatic mammals, which may range from harmful, neutral, and beneficial to the host (Soto et al., 2010; Urbanczyk et al., 2011). One particular mutualistic interaction is the partnership between marine bioluminescent *Vibrio* and sepiolid squid. The sepiolid squid-*Vibrio* symbiosis has been a model system for studying developmental biology, immunology, physiology, and molecular biology underpinning interactions between bacteria and animals for over two decades (McFall-Ngai and Ruby, 1991), since both partners can easily be maintained in the laboratory independently of each other. Sepiolid and loliginid squids (**Figure 1A**) are colonized by bioluminescent *Vibrio* (Fidopiastis et al., 1998; Guerrero-Ferreira and Nishiguchi, 2007). The bioluminescent bacteria inhabit a morphological structure called the light organ (**Figure 1B**) within the squid mantle cavity and benefit from their association with the cephalopod host by inhabiting a microenvironment rich in nutrients relative to the oceanic water column. The squid hosts prosper from the presence of bioluminescent bacteria by utilizing the light produced for a cryptic behavior called counterillumination (Jones and Nishiguchi, 2004; **Figure 1C**). Female squid fertilized by males lay their eggs on solid substrates such as rocks, where the embryos develop. Since female sepiolid squids are sequential egg layers, they can produce several clutches over 4–5 months after sexual maturity, with each clutch being 50–500 eggs each (Moltschaniwskyj, 2004). Axenic squid hatchlings emerge from their eggs (usually at twilight or at night) with sterile light organs, which are colonized within a few hours by specific free-living bioluminescent *Vibrio* present in the ocean (Soto et al., 2012). The colonizing bacteria quickly reproduce to fill and occupy the light organ crypt spaces (i.e., lumina, **Figure 1D**). Daily at dawn 90–95% of the light organ symbionts are vented exteriorly to the ocean by the squid host prior to burying in the sand. The remaining bacterial fraction in the squid host re-grows throughout the day to reinstate a complete light organ population by sunset (Soto et al., 2012). At dusk the squid emerge from the sand to engage in their nocturnal activity, including foraging and mating. [More detailed and comprehensive information can be found in recent reviews (Dunn, 2012; Stabb and Visick, 2013)]. Since bioluminescent symbionts can be grown in pure culture, cryopreserved with possible subsequent resuscitation, genetically manipulated and analyzed, and used to inoculate recently hatched gnotobiotic squid juveniles, the sepiolid squid-*Vibrio* mutualism is a promising prospect for experimental evolution studies aiming to understand symbioses. The juvenile squids are born without their *Vibrio* symbionts, lending the ability to infect the juveniles with any strain of *Vibrio* bacteria to examine colonization rates, ability to colonize, and persistence. Additionally, these bacteria can be used in competition experiments, which allows one to test different wild type strains against one another, mutant strains against their original wild type strain or versus other mutants, and experimentally evolved strains against the original ancestor (Nishiguchi et al., 1998; Nishiguchi, 2000, 2002; Soto et al., 2012).

**FIGURE 2:**
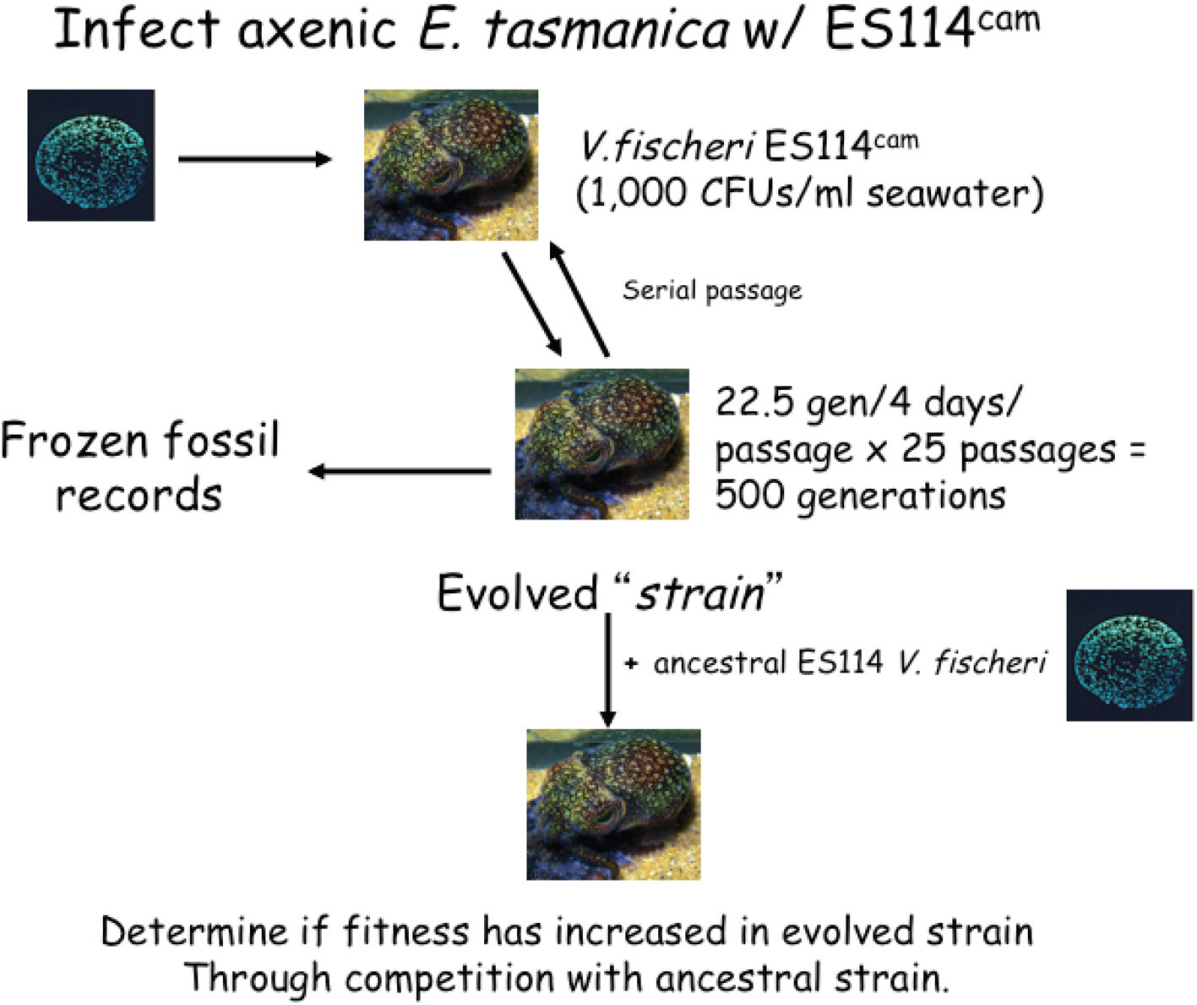
**Flow chart of experimental evolution studies in the Southern dumpling squid, *E. tasmanica*.** *Vibrio fischeri* ES114^CAM^ is a isogenic strain of *V. fischeri* ES114 (native to the Hawaiian bobtail squid, *E. scolopes*), with a neutral chloramphenicol-resistance marker (CAM). This strain was passaged through a non-native host for 500 generations to determine whether fitness would improve over each generation. Throughout the study, frozen fossil records can be made to eventually compare ancestor to evolved clones. Similar experiments can be completed in *E. scolopes*, including control animals (Soto et al., 2012).

Nevertheless, this mutualism has only recently been tapped as a resource for microbial experimental evolution studies in recent years (Schuster et al., 2010; Soto et al., 2012, 2014). Early work has shown *V. fischeri* are able to adapt to a novel squid host within 400 generations (**Table 1**), and such evolution may create tradeoffs in the ancestral animal host environment or in the free-living phase as a physiological correlated response to an important abiotic factor (Soto et al., 2012). Two sepiolid squid genera, *Euprymna* and *Sepiola*, are in the same taxonomic family. Several different *Euprymna* species are distributed allopatrically throughout the Indo-West Pacific Ocean, while numerous *Sepiola* species simultaneously co-occur sympatrically in the Mediterranean Sea (Nishiguchi et al., 1998; Nishiguchi, 2000, 2002; Soto et al., 2009). *Vibrio* symbionts colonizing *Euprymna* are host specialists and outcompete allochthonous isolates, a phenomenon termed competitive dominance, while those colonizing *Sepiola* are host generalists. *Vibrio* symbionts display no competitive dominance within *Sepiola* (Nishiguchi et al., 1998; Nishiguchi, 2000, 2002; Wollenberg and Ruby, 2012). Despite the presence of competitive dominance, data from population genetics and phylogenetics suggested secondary colonization events have occurred (Nishiguchi and Nair, 2003; Jones et al., 2006; Urbanczyk et al., 2011), creating a puzzling conundrum for years. Population genetics surveys fueled this enigma by consistently observing high levels of genetic diversity within the squid light organ (Jones et al., 2006), indicating light organ populations are not dominated by single or few genotypes through space and evolutionary time, an observation not consistent with competitive dominance. Competitive dominance results from squid host specialization by the symbionts, which should presumably purge genetic diversity of *V. fischeri* populations inside light organs. Microbial experimental evolution shed light on these mysteries and helped resolve these paradoxes with a complementing temporal population genetics survey spanning a decade—about 20,000 *V*. *fischeri* generations of evolution within the squid host—revealed the same evolutionary forces begetting competitive dominance were also responsible for driving *V. fischeri* genetic and phenotypic diversity within the squid light organ (Soto et al., 2012, 2014). *V. fischeri* indigenous to the Hawaiian bobtail squid (*E. scolopes*) was serially transferred for 500 generations through the Australian dumpling squid (*E. tasmanica*), a novel host (Soto et al., 2012; **Figure 2**).

**Table 1:** Competitive colonization experiments between *Vibrio fischeri* strains ES114 (ancestor) and JRM200 (derived) at different evolutionary time points in the novel squid host *Euprymna tasmanica*.

Evolutionary time point (Generations)	Expected ES114:JRM200 (Percentage ancestor: percentage evolved)	Observed ES114:JRM200 (Percentage ancestor: percentage evolved)
0 (n = 33)	50:50	46:54
100 (n = 24)	50:50	47:53
200 (n = 24)	50:50	41:59
300 (n = 24)	50:50	41:59
400 (n = 24)	50:50	35:65^1^
500 (n = 24)	50:50	36:64^1^

*^1^Significantly different two-tailed *t*-test and sign test (*P* < 0.05, α = 0.05)*.

Results demonstrated as *V. fischeri* adapted to *E. tasmanica*, the ability of the derived lines to grow along a salinity gradient significantly changed relative to the ancestor. Moreover, no obvious pattern to the growth changes was evident across the salinity continuum, suggesting *V. fischeri* microbial physiology had been “randomized.” Salinity is known to impact *Vibrio* population levels and distributions worldwide (Soto et al., 2009). *V. fischeri* subjected to novel host evolution created polymorphic reaction norms for salinity, an abiotic factor integral to shaping symbiont ecology during the free-living phase. Furthermore, experiments indicated a “superior numbers” or a “running start” advantage to foreign strains over native ones in animal host colonization that could outflank competitive dominance. Thus, *V. fischeri* strains most abundant (perhaps due to salinity) during the free-living phase where squid hosts resided were the ones most likely to colonize the cephalopod, not strains best adapted to the squid (Soto et al., 2012). A similar process may occur with *Photobacterium* in fish hosts due to temperature (Urbanczyk et al., 2011). Additionally, the *V. fischeri* lines serially passaged through *E. tasmanica* surged in biofilm formation and bioluminescence but lessened in motility (Soto et al., 2014). Increases and decreases in the utilization of select carbon sources also transpired. Interestingly, evolutionary differentiation occurred in the derived lines relative to the ancestor and to each other for biofilm formation, motility, bioluminescence, and carbon source metabolism, results consistent when compared to *V. fischeri* wild isolates obtained from light organs of *E. scolopes* and *E. tasmanica* specimens collected in the field (Soto et al., 2014). Squid host specialization by the symbionts promotes competitive dominance and diversifying selection. Perhaps clonal interference prevents selective sweeps in the squid light organ. The lineages serially transferred through *E. tasmanica* also exhibited decreased levels of bioluminescence in the ancestral host *E. scolopes* (Soto et al., 2012). In an independent study, *V. fischeri* strains previously incapable of establishing a persistent association (chronic infection) with sepiolid squids were shown to be capable of doing so after serial passage in *E. scolopes* (Schuster et al., 2010). Since *V. fischeri* possesses a life history where bacteria are cyclically associated with an animal host (sepiolid squids and monocentrid fishes) and then outside the host as free-living bacteria in the ocean, researchers can use microbial selection experiments with *V. fischeri* to simultaneously study symbiosis evolution and microbial evolution in the natural environment where microbes are not partnered to a host. [Some recent work suggests *V. fischeri* may also be a bioluminescent symbiont in the light organs of fishes belonging to the taxonomical families Moridae and Macrouridae (Urbanczyk et al., 2011).] Additionally, *V. fischeri* strains exist which are completely unable to colonize the light organs of sepiolid squids and monocentrid fishes, permitting evolutionary biologists to study a continuum of interactions between a microbe and animal host when studying the squid-*Vibrio* mutualism. Given the Sepiolidae is a diverse family of squids that include allopatric and sympatric species distributions, testing whether host speciation affects selection for host specialist versus host generalist evolutionary strategies within *Vibrio* symbionts is possible.

### TYPE STRAIN MENTALITY AND OTHER BIOLUMINESCENT SYMBIONTS FOR SEPIOLID SQUIDS

Early work characterizing the molecular biology of *V. fischeri* colonizing *Euprymna* squid focused on the strain *V. fischeri* ES114 and the host *E. scolopes* (with occasional studies in *Sepiola*), since only the Hawaiian squid host was routinely available (McFall-Ngai and Ruby, 1991; Fidopiastis et al., 1998). Furthermore, reductionism was desired to understand the fundamentals of the symbiosis. Nonetheless, caution is warranted to avoid development of a “type strain” or “type host” mentality. Recent work has expanded to regularly include other strains of *V. fischeri* and *Euprymna* species (Ariyakumar and Nishiguchi, 2009; Chavez-Dozal et al., 2012; Soto et al., 2012). This will aid in identifying more general results from those that are specific to a particular symbiont strain or host species. In addition, initial characterization of the sepiolid squid-*Vibrio* symbiosis described *V. fischeri* as the only bioluminescent symbiont present in the squid light organ (McFall-Ngai and Ruby, 1991). Subsequently, *V. logei* was discovered as a symbiont in the genus *Sepiola* (Fidopiastis et al., 1998; Nishiguchi, 2000). More recently, *V. harveyi* and *Photobacterium leiognathi* have been included as symbionts of *E. hyllebergi* and *E. albatrossae* from Thailand and the Philippines, respectively (Guerrero-Ferreira and Nishiguchi, 2007; Guerrero-Ferreira et al., 2013). An important prospect to consider is that *V. fischeri* and *V. logei* may have evolved fundamentally distinct and different traits for colonizing sepiolid squids, even when considering the same host species. Clearly, new and thrilling perspectives are surfacing around the sepiolid squid-*Vibrio* mutualism. Several species in the Vibrionaceae are bioluminescent. An interesting remaining question is why only a few of these form light organ symbioses with sepiolid squid hosts. For example, why is bioluminescent *V. orientalis* never found in squid light organs (Dunlap, 2009)? Are researchers simply not looking thoroughly enough?

### BIOGEOGRAPHY OF *VIBRIO* BACTERIA AND EXPERIMENTAL EVOLUTION IN THE FIELD

Experimental evolution in the lab with *Vibrio* bacteria has only been completed in one species of *Vibrio* (*V. fischeri*), and strains used in those studies were either from the squid host *E. scolopes* (Hawaii) or pinecone fish *Monocentris japonicas* (Schuster et al., 2010; Soto et al., 2012). Given that a number of symbiotic *V. fischeri* from squid can colonize and survive in nearly all allopatric *Euprymna* hosts of the Indo-West Pacific, it provides a road map whether other *V. fischeri* strains can adapt to additional potential host species closely related to *Euprymna* (e.g., *Rondeletiola minor*) or even ones from a different phylum (Nishiguchi et al., 2004). Naturally occurring strains may be subjected to movement between hosts that are along a specific environmental gradient (Soto et al., 2010). Obviously, similar cues must be used for these bacteria to recognize a comparable, yet novel host, and then colonize and establish a persistent association in the outré animal for the symbionts to secure their distribution in the new host population (Wollenberg and Ruby, 2009). Only 6–12 *V. fischeri* cells are required to initiate a squid light organ infection. Once these bacteria colonize a squid host, they can reproduce much faster than in seawater. New *V. fischeri* clones encountering a squid host species for the first time will then be expelled every 24 h, increasing the cell numbers of *V. fischeri* new arrivals that can infect even more juvenile squid of the exotic host species (Lee and Ruby, 1994b, 1995). Whether these symbiont founder flushes truly occur in nature is not known, but observations in the laboratory have shown that alien *V. fischeri* genotypes can invade and take root where a preexisting genetic variety was already entrenched (Lee and Ruby, 1994a; Soto et al., 2012). Whether this commonly leads to a dominant symbiont genotype in a host population in a given geographical area over the long term must be investigated more closely.

## TWO-CHROMOSOME GENOMIC ARCHITECTURE IN VIBRIONACEAE, EVOLVABILITY, AND VERSATILITY

Research has shown an absence of parallel coevolution between *V. fischeri* symbionts and their light organ animal hosts, which implies significant host switching has occurred (Nishiguchi and Nair, 2003). Host switching has been a common evolutionary phenomenon for *Vibrio* and *Photobacterium* species involved in symbioses, regardless of whether the interaction was commensalism, pathogenesis, or mutualism (Urbanczyk et al., 2011). Extensive host switching could suggest this microbe, along with *Vibrio* species in general, are evolutionarily plastic and malleable organisms. Vibrionaceae possess two circular chromosomes, one large (Chromosome I) and one small (Chromosome II; Tagomori et al., 2002). With this complex genome arrangement, *V. fischeri*’s ability to exploit numerous lifestyles is easy to understand, as the *Vibrio* genome structure is dynamically unstable (Kolsto, 1999). The modular two-chromosome architectural structure of Vibrionaceae genomes has been hypothesized to be the inception for the versatility and ubiquity of this cosmopolitan bacterial family, with ecological specialization being the essence of the smaller and more genetically diverse Chromosome II with its superintegron island gene-capture system and genes encoding for solute transport and chemotaxis (Heidelberg et al., 2000; Ruby et al., 2005; Grimes et al., 2009). Intrachromosomal and interchromosomal recombination is clearly present, along with inversions, indels, and rearrangements (Kolsto, 1999; Heidelberg et al., 2000; Tagomori et al., 2002). Such genomic architecture permits the evolutionary potential for functional genetic specialization to occur among the two chromosomes (Heidelberg et al., 2000; Waldor and RayChaudhuri, 2000), promoting ecological opportunity in adapting and radiating into numerous niches (Soto et al., 2014). For example, *V. cholerae* and *V. parahaemolyticus* genomic studies have discovered that house-keeping genes (DNA replication, transcription, translation, cell division, and cell wall synthesis) and pathogenicity are mainly restricted to the large chromosome (Heidelberg et al., 2000).

Chromosome II appears to be a genetic module for DNA and a source for innovation, perhaps evolutionarily functioning analogous to plasmids, possessing significantly more foreign loci that appear to have been acquired horizontally from other microbial taxa (Heidelberg et al., 2000; Waldor and RayChaudhuri, 2000). The presence of a gene capture system (i.e., integron island) and genes usually found on plasmids support this claim (Heidelberg et al., 2000). Furthermore, loci involved in substrate transport, energy metabolism, two-component signal transduction, and DNA repair are prominently carried on Chromosome II (Heidelberg et al., 2000; Waldor and RayChaudhuri, 2000). The loci involved in substrate transport consist of a large repertoire of proteins with diverse substrate specificity. Genes that subdivide cellular functions and that are intermediaries of metabolic pathways also are found on Chromosome II. These genetic auxiliaries potentially serve as the raw material for adaptation and specialization (Heidelberg et al., 2000; Waldor and RayChaudhuri, 2000). The structure and size of the large chromosome appears relatively constant throughout the Vibrionaceae, whereas Chromosome II is more acquiescent and flexible to genetic reorganization, rearrangement, recombination, and large indel events (Okada et al., 2005). Genes encoding function for starvation survival and quorum sensing are located on both chromosomes. Thus, interchromosomal functional regulation is present in Vibrionaceae. As a result, specific and novel mechanisms involved in the regulation, replication, and segregation of both chromosomes are thought to have evolved in this bacterial family (Waldor and RayChaudhuri, 2000; Egan et al., 2005).

Interestingly, *V. cholerae* colonization factors (e.g., genes responsible for pili formation) primarily reside on Chromosome I. Consequently, different *V. fischeri* ecotypes could be the result of evolution at loci involved in metabolism as opposed to those involved in tissue colonization (Browne-Silva and Nishiguchi, 2007; Soto et al., 2014). Experimental evolution studies with *E. coli* have demonstrated that resource partitioning and alternative substrate specialization is sufficient for ecological polymorphisms to arise in prokaryotes (Rosenzweig et al., 1994). In summary, the two-chromosome architecture provides *V. fischeri* with enormous evolutionary fluidity. Particularly, Chromosome II may possess ecological or symbiosis islands which could account for this microorganism’s broad ecological range (Tagomori et al., 2002). For example, differences in the pathogenicity islands present on Chromosome II appear to determine whether or not *V. parahaemolyticus* strains are pathogenic. Similarly, the pliant nature of *V. fischeri* could explain why there is extensive host switching. Chromosome II may well be a gene repository outfitted to respond to environmental change, habitat heterogeneity through space and time, and stress (Dryselius et al., 2007; Soto et al., 2012). Future studies will be thrilling and exciting, as modern bioinformatics and genomics offer high hopes and allow unprecedented visions. Recent advances in high throughput sequencing technologies and genome editing techniques (e.g., MuGENT) will greatly increase the potential of experimental evolution to understand adaptation (MacLean et al., 2009; Dalia et al., 2014).

## TOPICS FOR FUTURE STUDY

### BIOFILM FORMATION AND MOTILITY

**FIGURE 3:**
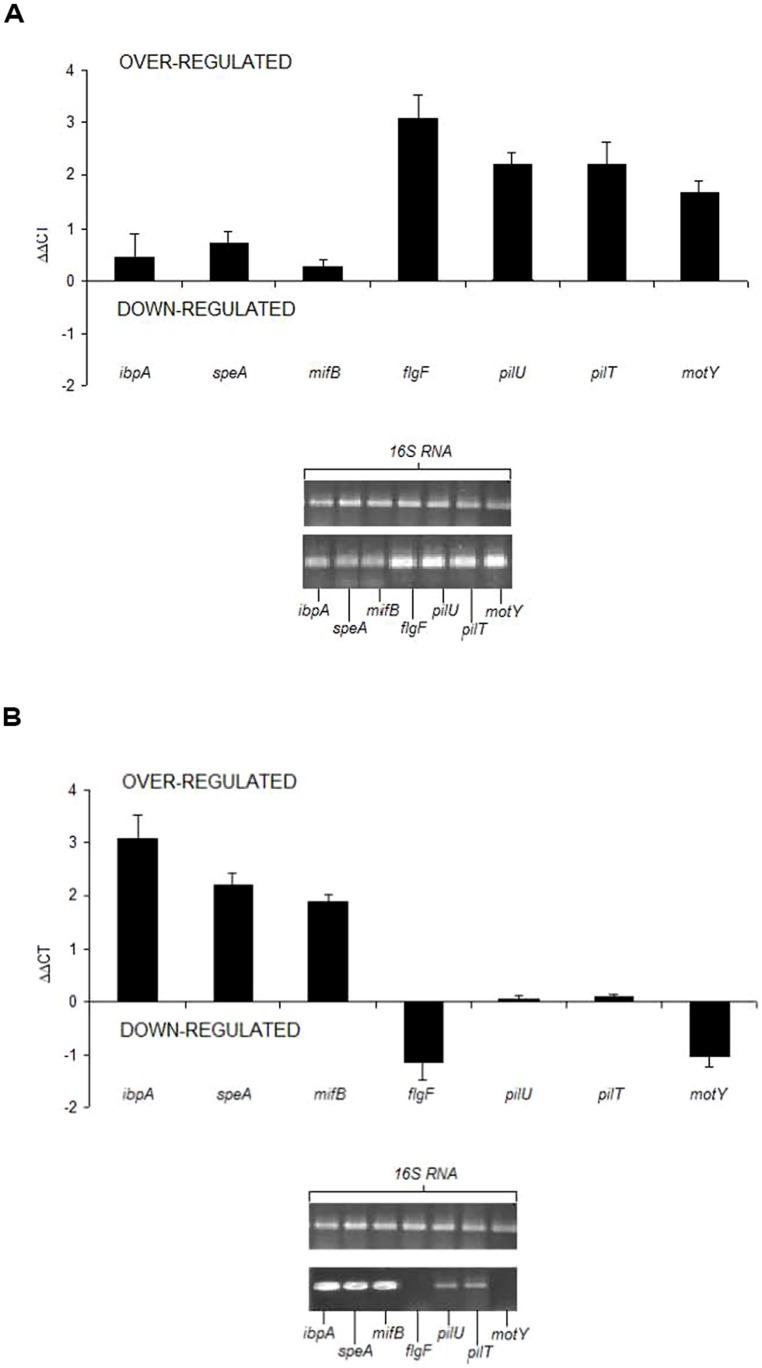
**Early and late gene expression (mRNA) of various biofilm related loci in *V. fischeri* ETJB1H. (A)** Early (4 h) gene expression of flagellum biosynthesis (*flgF*), type IV pili formation and adhesion (*pilU, pilT*), and the sodium-type flagellar motor pump for motility (*motY*) loci. **(B)** Late gene expression of genes important for mature biofilm production (24 h) include expression of heat shock protein (*ibpA*), magnesium-dependent induction for c-di-GMP synthesis (*mifB*), and arginine decarboxylase (*speA*). Modified from Chavez-Dozal et al. (2012). dCCT is the change in relative expression of each gene compared to the standard control (in this case 16S rRNA). The gel represents the amount of 16S rRNA (top) and the amount of mRNA expressed in each gene examined. Error bars represent SD of three replicates.

Motility and biofilms are modes by which *V. fischeri* strains can niche specialize in their *Euprymna* squid hosts (Yildiz and Visick, 2009; Soto et al., 2014). Biofilms are aggregates of microorganisms attached to a surface that are frequently enmeshed within a matrix of exopolysaccharide and can be comprised of a pure culture population or a community (Davey and O’toole, 2000; Stoodley et al., 2002). This community is much more resistant to antimicrobials, ultraviolet light, pH shifts, osmotic shock, desiccation, and other environmental stresses (Gilbert et al., 1997; Davey and O’toole, 2000). The role of biofilms in disease and host colonization is well documented, where bacterial pathogens establishing biofilms in animals may be more recalcitrant to phagocytosis by host macrophages, resistant to respiratory bursts by immune cells, and insensitive to antimicrobials produced by host defenses (Davey and O’toole, 2000). In addition, biofilm development has a major role in *V. fischeri* colonization of sepiolid squid hosts (Chavez-Dozal and Nishiguchi, 2011; Chavez-Dozal et al., 2012; **Figure 3**). When movement on surfaces is necessary, swarming with flagella is the motility mechanism for Vibrionaceae (McCarter, 2001). Swarming is specialized mobilization or locomotion on a surface as opposed to the swimming and tumbling done by individual cells. As *V. fischeri* swarms with concurrent cell division (e.g., growth), cells differentiate from a vegetative state to a swarmer one. Swarmer cells are hyperflagellated and longer than vegetative counterparts (Harshey, 2003), and provide a steady state supply of nutrients until motility ceases. Motility plays an integral role in the colonization of sepiolid squid by *V. fischeri* and allows host-associated bacteria to reach the destination and surface desired for further colonization or attachment (Millikan and Ruby, 2004). Since swarming is an energetically expensive process, chemotaxis has a role mediating how a bacterial cell should physiologically respond. Through years of studying diverse bacteria as motility model systems, research has shown many regulatory pathways controlling motility also affect biofilm formation (Harshey, 2003; Verstraeten et al., 2008). Bacterial populations must resolve whether to institute motile machinery for expedient colonization of surfaces or engage biofilm systems when an appropriate location for initial contact and attachment has been found, a critical choice affecting survival between competitors. Experiments are underway where *V. fischeri* lines are being selected for increased biofilm formation and motility. Accompanying these experiments are ones where *V. fischeri* lines are being alternately or cyclically selected for biofilm and motility lifestyles (oscillatory selection). The relative abilities of these lines to colonize squid hosts will be assessed.

### PARASITISM, PREDATION, AND GRAZING ON *VIBRIO* BACTERIA

Substantial work exists on how protoctistan predators are effective grazers on *Vibrio* or other bacteria, particularly when they form biofilms (Matz and Kjelleberg, 2005; Matz et al., 2008). Previous research has demonstrated that certain species of *Vibrio* (e.g., *V. cholerae* and *V. fischeri*) are better able to ward off microbial eukaryotic predators when in their biofilm state compared to their planktonic counterparts (Erken et al., 2011). Earlier work provides strong evidence that when *Vibrio* biofilms are grazed by protoctistans, the bacteria release toxic compounds capable of killing the predators, the dead grazers themselves then become a meal and carbon source for the *Vibrio* (Chavez-Dozal et al., 2013). Depending on the species, and even strain type, *Vibrio* biofilms make an excellent model to determine if grazing can affect biofilm growth, structure, and production of chemicals to inhibit grazers (Barker and Brown, 1994). Current microbial selection studies are ongoing to examine adaptive responses of *V. fischeri* to various grazers and how these evolutionary outcomes impact sepiolid squid colonization. Bacteriophage, predatory bacteria (e.g., *Bdellovibrio*, *Bacteriovorax*, *Micavibrio*, and “wolfpack” feeders such as myxobacteria), and aquatic fungi also prey on the Vibrionaceae (Atlas and Bartha, 1998; Richards et al., 2012). How these natural enemies affect *V. fischeri* evolution and the sepiolid squid-*Vibrio* symbiosis are worthy of future investigations. For instance, *Vibrio* chitinases attacking fungal cell walls may be a means to avoid grazing by marine yeast. Chitinases are known to be utilized by *V. fischeri* symbionts when interacting with the squid host (Wier et al., 2010).

### EVOLUTION DURING THE FREE-LIVING PHASE, ABIOTIC FACTORS, AND BACTERIAL STRESS RESPONSES

Prior work has shown that *V. fischeri* host adaptation to sepiolid squids and monocentrid fishes affects this species’ ability to grow within a gradient of an abiotic factor (e.g., tolerance limits to environmental stress) while in the free-living or planktonic phase (Soto et al., 2009, 2012), implicating that natural selection could be acting on the bacterial stress responses to better accommodate the symbiont against the unprecedented stressful environments presented by a new animal host (e.g., novel immune defenses; Soto et al., 2010). The coupling of different bacterial stress responses to one another and their correlation to successful symbiosis initiation, host immunity evasion, pathogenesis, and virulence mechanisms is becoming necessary for understanding bacterial evolution (Nishiguchi et al., 2008). Future experimental evolution work will focus on the adaptability of *V. fischeri* to abiotic factor stresses, such as high and low tolerance limits of salinity, temperature, and pH while in the free-living or planktonic phase. In turn, correlated responses of *V. fischeri* adapting to these environmental stresses will be investigated in sepiolid squid hosts (Abucayon et al., 2014). Understanding how *V. fischeri* stress evolution affects its relationship with sepiolid squids will lead to new insights in the dynamic evolutionary forces that shape associations between hosts and symbionts. Because both free-living and host environments impose dramatically different selection pressures to microorganisms (e.g., evasion of immune host defenses), these perspectives have implications into infectious disease and virulence mechanisms, as genetic and physiological components responsible for mutualisms and pathogenesis are frequently identical or homologous (Ruby et al., 2005; Nishiguchi et al., 2008; Buckling et al., 2009). Stress evolution and stress-induced mutagenesis are known to be capable of creating cryptic genetic variation through varying gene-by-gene and gene-by-environment interactions which can be invisible to natural selection during the original circumstances in which they materialize but either beneficial or detrimental to bacterial fitness when conditions change (Tenaillon et al., 2004; McGuigan and Sgro, 2009; Paaby and Rockman, 2014). The evolutionary significance of cryptic genetic variation in patterning interactions between animal hosts and bacteria is unclear. *V. fischeri* adapting to a novel squid host was found to increase this symbiont’s ability to form biofilms in artificial seawater containing no organic carbon while in the free-living phase. This result suggests symbiosis evolution can affect *V. fischeri*’s ability to tolerate starvation or oligotrophic conditions when subsequently outside the host (Soto et al., 2014). *V. fischeri* adapting to selective pressures imposed by abiotic factors or environmental stressors during the free-living phase may either reinforce or decouple coevolution between *Vibrio* symbionts and their animal hosts (Soto et al., 2009, 2012). A static microcosm or standing culture of *Pseudomonas fluorescens* where wrinkly spreader, fuzzy spreader, and smooth morph colonies arise over several days has become a model system for studying microbial adaptive radiation, a process known to be affected by oxygen depletion and nutrient availability (Rainey and Travisano, 1998; Travisano and Rainey, 2000). Alterations in *Vibrio* colony morphology is known to affect animal host colonization (Mandel et al., 2009). *V. fischeri* adaptive radiation during the free-living phase and the subsequent consequences on symbiosis are poorly understood. The use of microbial experimental evolution with heterogeneous environments will provide insight into how *V. fischeri* biodiversity (e.g., Shannon-Wiener Index) in the free-living phase affects symbiont population variation within the squid light organ across gradients of various abiotic factors.

### METABOLISM

Biolog plates were developed for global phenotype analysis of microorganisms that allows a comprehensive survey of microbial physiological traits (Bochner, 1989; Bochner et al., 2001). The aim is to identify unique characteristics of individual microbes and common metabolism to particular taxa or ecological populations. These plates also provide functional data to complement genetic analyses and gene expression studies of microbes. For instance, mutants can be screened efficiently to compare phenotypic consequences relative to wild type. This is especially important for examining metabolic polymorphisms, physiological heterogeneity, and distinguishing between different ecotypes within the same bacterial species, since different substrates can be shunted into alternate biochemical pathways (Rosenzweig et al., 1994; White, 2007). Additionally, how metabolism of the same substrate (i.e., D-glucose) is disproportionately distributed among numerous biochemical pathways (glycolysis versus pentose phosphate pathway) may also vary among different individual cells of the same bacterial species (Rosenzweig et al., 1994), as hypothesized by the nano-niche model of bacterial evolution (Wiedenbeck and Cohan, 2011). For example, most members of an *E. coli* population may move the carbon flow from the breakdown of D-glucose via glycolysis, but a small proportion of the remaining population may shuttle more intermediates of D-glucose degradation through Entner-Doudoroff pathway for an alternate way of making a living (e.g., physiological tradeoffs, resource partitioning, and ecological nutrient specialization by differentiation in usage of metabolic pathways; Rosenzweig et al., 1994; Cooper and Lenski, 2000; Travisano and Rainey, 2000; MacLean et al., 2004; White, 2007). Within the lifetime of just one adult squid host, a single *V. fischeri* clone has ample time to evolve cross-feeding with either other *V. fischeri* cells or host cells, since this has been documented in *E. coli* in less than 800 generations in a homogenous and unstructured environment (Rosenzweig et al., 1994). *V. fischeri* adapting to novel animal hosts undergo ecological diversification in carbon source utilization within 500 generations (Soto et al., 2014). With the use of Biolog plates, microbial experimental evolution can provide keen insight in the role of metabolism in *V. fischeri* ecological diversification and sepiolid squid colonization (MacLean and Bell, 2002).

### CHEMOTAXIS

Support exists biofilms, motility, carbon metabolism, and bioluminescence are entwined or interlaced with one another. Possible crossroads for their roles in *V. fischeri* include chemotaxis, intracellular second messengers (c-di-GMP), and bacterial stress responses. Methyl-accepting chemotaxis proteins (MCPs) are central for chemotaxis, as these proteins are chemoreceptors that monitor the chemical composition of the environment and transmit this information interiorly to the cell (Bren and Eisenbach, 2000; Brennan et al., 2013). MCPs are versatile receptors to chemical stimuli, adept at mediating taxis to diverse signals (Hsing and Canale-Parola, 1996). A single MCP is incredibly sensitive. It is able to discern differences in stereochemistry between isomers, sense relative asymmetries in chemical concentrations of a substance along a gradient, and integrate diverse information of multiple chemical stimuli present in the environment simultaneously (Hsing and Canale-Parola, 1996; Bren and Eisenbach, 2000). An MCP is capable of a graded, measured, and progressive selective response to chemical stimuli. MCP function is further elaborated by being present on bacterial cell membranes as a mass complex of several interacting MCPs bundled together into a chemo-antenna cluster network, amplifying the synergistic interactions possible in chemotaxis and signal transduction (Bren and Eisenbach, 2000). Additionally, single amino acid substitutions can have colossal effects in sensitivity, affinity, specificity, and function of an MCP (Derr et al., 2006). Hence, MCPs and redistributable metabolism may allow *V. fischeri* populations to better colonize novel hosts by resculpting its N-dimensional niche hypervolume space quickly (Hutchinson, 1957). In a study using comparative genomics and a network biology-based approach to understand how genes select for multigenic phenotypes such as virulence in *V. cholerae*, loci encoding MCPs and others associated with chemotaxis were among those identified as most responsible (Gu et al., 2009). MCPs couple chemotaxis to diverse metabolites and their gradients, supplying one potential route a symbiont can adapt to unaccustomed host physiology. Experimental evolution with microorganisms to analyze chemotaxis can be completed by placing small volumes of bacteria onto the centers of motility agar plates with different chemoattractants at the periphery. Over an incubation time at an appropriate temperature, cells from the leading edge closest to the chemoattractant are serially transferred onto the centers of new motility plates (DeLoney-Marino et al., 2003). Derivations of this method can be used to select for bacteria with increased aversion to chemorepellents. Another avenue is to use a rendition of the glass capillary tube chemotaxis assay that involves continuous subculturing (Adler, 1973).

### QUORUM SENSING, BIOLUMINESCENCE, SOCIAL EVOLUTION, AND ECOLOGICAL INTERACTIONS

Quorum sensing was first described in *V. fischeri* in 1970 in connection with bioluminescence (Nealson et al., 1970). Since then, quorum sensing is now known to govern many more traits other than bioluminescence, including but not limited to exoenzyme secretion, siderophore production, antibiotic synthesis, cell division, DNA replication, cell surface anabolism (cell wall, cell envelope, and capsule), biofilm development, and motility (Miller and Bassler, 2001). Bioluminescence is frequently used as a proxy quorum sensing measurement. Regulation of the *lux* operon involves input from the quorum sensing apparatus that couples to other microbial physiological pathways and cascades (Miyashiro and Ruby, 2012). Clever designs can permit microbial selection experiments that investigate quorum sensing and bioluminescence. In a plate selection scheme, ImageJ (image processing freeware produced by National Institutes of Health) may be used to single out brighter and dimmer colonies on agar plates for serial transfers that have been digitally imaged in lit and dark rooms (“digital replica plating” or “replica imaging”). The Vibrionaceae possess a hierarchical and sophisticated quorum sensing machinery comprised of “low cell density” (LCD) and “high cell density” (HCD) gene expressions (Camara et al., 2002). Microbial selection experiments with *V. fischeri* mutants locked or defaulted into LCD and HCD gene expressions will permit studies into group selection, kin selection, social evolution, and greenbeard genes (Travisano and Velicer, 2004). LCD and HCD gene expressions can each secrete a different and distinct subset of public goods not produced by the other (e.g., extracellular nuclease and metalloprotease for LCD and HCD, respectively; Blokesch and Schoolnik, 2008; Natrah et al., 2011; Bruger and Waters, 2014). Experimentally evolved lines possessing constitutive HCD and LCD gene expressions would be compared to the quorum sensing wild type strain (ancestral or derived) for a particular selection regimen. LCD lines could serve as “cheaters” or “defectors” for a public good produced by HCD or wild type lines at high cell density (e.g., extracellular metalloproteases). An investigator could ask if an LCD cheater line initially at low frequency could invade an HCD line (at low or high cell density) or a quorum sensing wild type line at high cell density. HCD lines could analogously serve as cheaters for extracellular nuclease. The ability to control microbial growth and dilution rates with chemostats using select media might also be another way. The use of quorum sensing enhancers and quorum quenching molecules or drugs are additional avenues for future experiments (Rasmussen and Givskov, 2006; Defoirdt et al., 2008). Serial transfers of liquid cultures performed at particular cell densities (specific transmission bottlenecks) or with spent (conditioned) media may permit inquiries into quorum sensing.

Other possible ecological interactions between microbes include competition (interference and exploitation) and microbial allelopathy (e.g., chemical warfare; Atlas and Bartha, 1998). Additionally, one must recognize that one microbe may be more fit than another because of increased efficiency in resource utilization or better able to convert assimilatory carbon and reducing power into more offspring (i.e., a shorter generation time growing on D-glucose). Yet an interesting facilitation is cross-feeding. Cross-feeding can also occur between cells of different strains or species, where one cell type secretes a waste product that is utilized by another as a nutrient or useful resource. Understanding the diversity of social dynamics is valuable. Within the social evolution context, when a participant (the actor) benefits from harming another (recipient), the interaction is termed selfishness (West et al., 2006). When the actor suffers a negative effect by harming the recipient, the interaction is called spite. Altruism occurs when the recipient benefits and the actor is harmed, but mutualism takes place with both partners benefiting. Commensalism occurs if the actor benefits and the recipient experiences no effect. In amensalism, the actor is unaffected but the recipient is disserviced (Atlas and Bartha, 1998). (Predation was addressed previously.) As alluded to earlier, an initial effort to characterize the assortment of social interactions between bacteria can be done by placing washed cells in the filter-sterilized spent media of a competitor. NMR and mass spectroscopy can possibly be used to identify any interesting molecular components that can be isolated or purified. Excellent questions linger. What are the roles of cooperation, cheating, competition for limiting resources, microbial allelopathy, and other ecological interactions in shaping the squid-*Vibrio* symbiosis? At what stages do each of these processes most predominate (e.g., free-living versus host associated)? Is cheating among symbionts suppressed by the squid host when *V. fischeri* are in the light organ? Are bacteriocins produced by *V. fischeri* strains (i.e., vibriocins) against other conspecific subtypes in the squid light organ?

### VIABLE BUT NON-CULTURABLE STATE

The viable but non-culturable (VBNC) state is a phenomenon frequently observed in the Vibrionaceae and other prokaryotes, including *V. fischeri* (Lee and Ruby, 1995). Bacteria normally culturable no longer grow in liquid culture or on agar media, because the cells enter a dormancy where still metabolically active and presumed to have elevated tolerance or resistance to environmental stressors (extreme conditions of an abiotic factor such as temperature or salinity), harmful compounds or noxious chemicals, starvation, and heavy metal toxicity (Ordax et al., 2006; Nowakowska and Oliver, 2013). Escape from digestion after phagocytosis or endocytosis by ameba and macrophages has also been hypothesized to be another function of the VBNC condition, permitting these eukaryotic cells to serve as reservoirs for survival and dispersal (Rahman et al., 2008). Published research has reported molecules and mechanisms (e.g., temperature upshift) that appear to restore culturability to VBNC cells upon their return to liquid media or agar plates. This putative revival of VBNC dormancy has been termed “resuscitation.” However, many researchers doubt the existence of a VBNC state and its resuscitation, claiming the supporting evidence is lacking or marginal at best (Bogosian and Bourneuf, 2001). Skepticism arises because resuscitation is thought to be re-growth of injured cells that have regained their health. Disbelievers point out genes responsible for a pathway or developmental program leading to a physiologically differentiated VBNC state have been slow to identify through the use of null mutations and knockout studies (Soto et al., 2010). Nothing analogous to endospore formation has surfaced. Definitive evidence of VBNC cells will require loss-of-function experiments with subsequent complementation or overexpression gain-of-function studies to describe a “VBNC” regulon or modulon (Bogosian and Bourneuf, 2001). Microbial experimental evolution is a remarkable approach to addressing the validity of VBNC cells. After 24–48 h of growth in nutrient rich media (28°C, 200–225 rpm), most of a *V. fischeri* liquid culture is non-culturable, if not entirely dead, as the plating efficiency rapidly decreases. (Static liquid cultures do not experience this phenomenon and can remain culturable for weeks). The exact result is strain dependent, as some strains are more susceptible than others in their failure to re-grow upon subculturing to fresh media or transfer to agar plates. By serially transferring what few *V. fischeri* cells continue to grow from shaking and aging liquid cultures undergoing a decay in culturability, a population can be increasingly selected for resistance to non-culturability.

## CONCLUSION

Bioinformatics will provide additional insight into experimental evolution with the Vibrionaceae, including genomics, transcriptomics, proteomics, and metabolomics. For microorganisms such as *V. fischeri*, which cycle between host-associated and free-living phases, consideration of the operating selection pressures unique to each environment, relative magnitudes, and respective contributions in driving microbial evolution merits consideration (Nyholm and Nishiguchi, 2008). Since prokaryotes possess tremendous genetic and metabolic diversity, understanding the factors that shape bacterial biogeography and ecology will provide insights into bacterial adaptation and natural history.

## Conflict of Interest Statement

The authors declare that the research was conducted in the absence of any commercial or financial relationships that could be construed as a potential conflict of interest.
